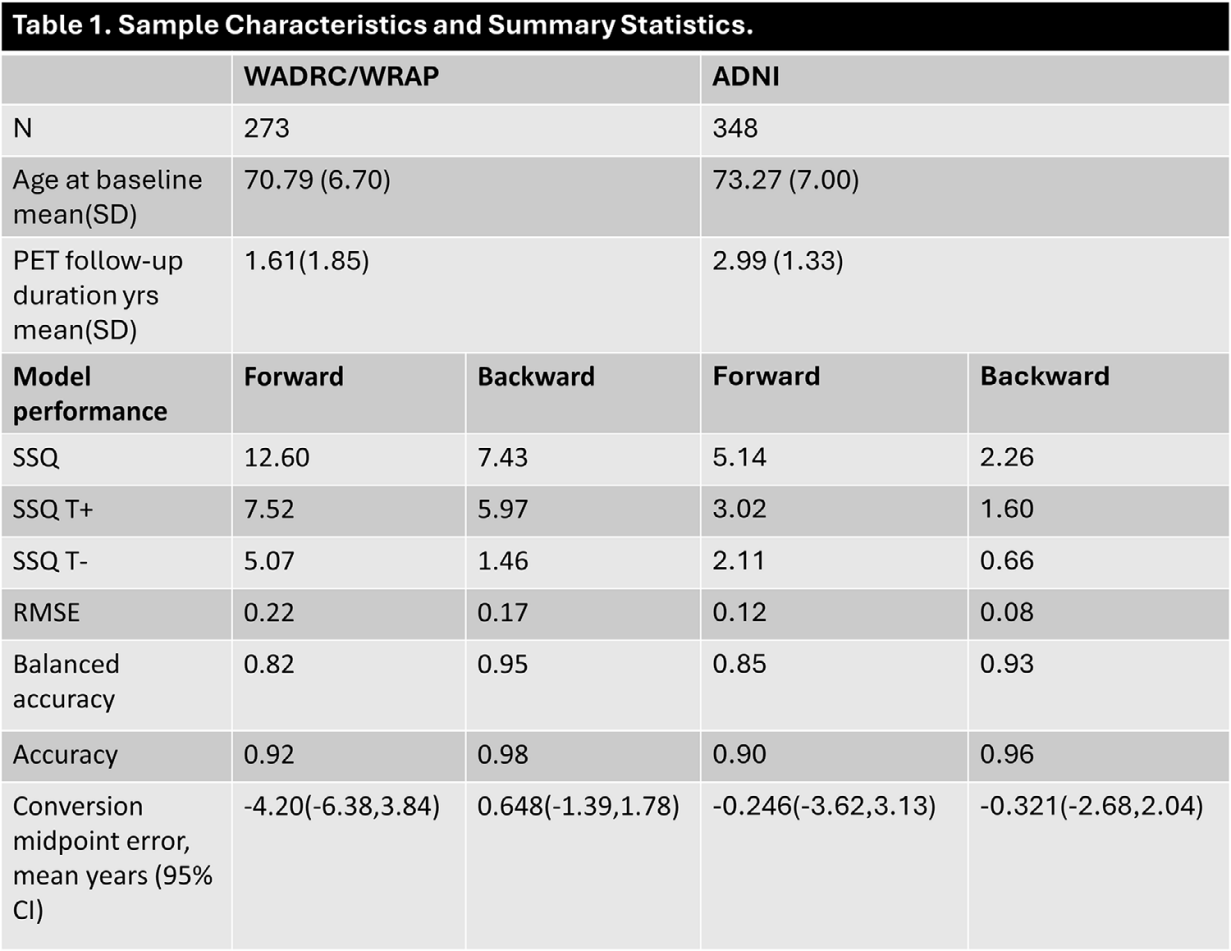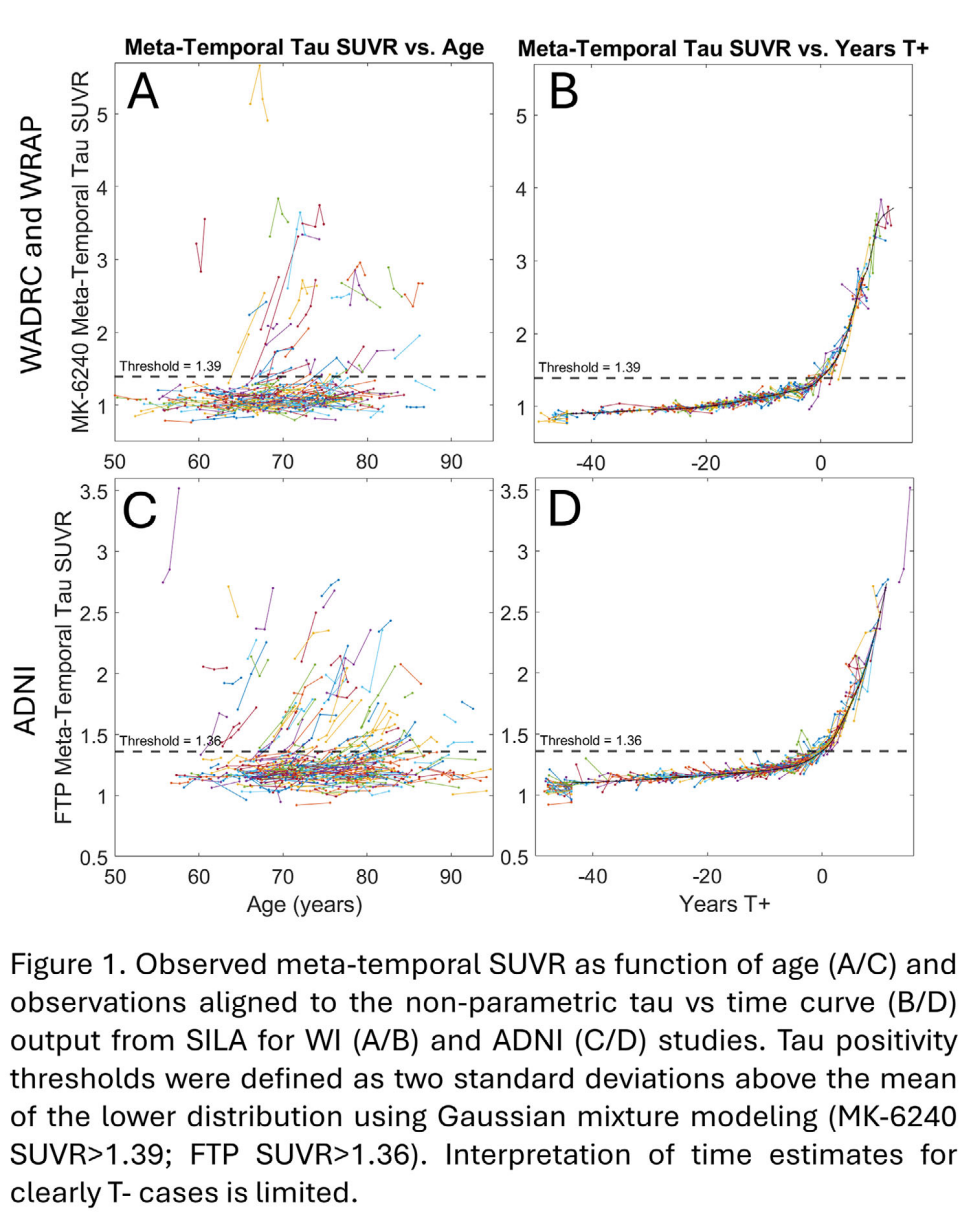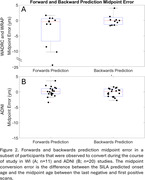# Multi‐cohort temporal modeling of tau PET onset age and trajectories

**DOI:** 10.1002/alz.093990

**Published:** 2025-01-09

**Authors:** Jordan P Teague, Margo B. Heston, Elena Ruiz De Chavez, Ruvini Navaratna, Karly Alex Cody, Jacob Morse, Yuetiva Deming, Bradley T. Christian, Sterling C. Johnson, Rebecca E. Langhough, Tobey J. Betthauser

**Affiliations:** ^1^ University of Wisconsin‐Madison School of Medicine and Public Health, Madison, WI USA; ^2^ Stanford University School of Medicine, Stanford, CA USA

## Abstract

**Background:**

Sampled iterative local approximation (SILA) is a temporal modeling method previously validated in three cohorts to estimate person‐level amyloid PET onset age and predicted change. This study validates SILA to model longitudinal tau PET trajectories, generate person‐level estimated tau onset age (ETOA), and characterize forward and backward prediction accuracy in the Wisconsin Registry for Alzheimer’s Prevention (WRAP), Wisconsin Alzheimer’s Disease Research Center (WADRC), and the Alzheimer’s Disease Neuroimaging Initiative (ADNI).

**Method:**

N=273 participants (mean(SD) baseline age=70.79(6.70)) from WRAP and WADRC underwent longitudinal 18 F‐MK‐6240 tau PET imaging. Data processing included Harvard‐Oxford ROI parcellation of T1‐weighted MRI and generation of regional MK‐6240 standard uptake volume ratios (SUVR; inferior cerebellar GM reference region; 70‐90 min). Volume‐weighted SUVR corresponding to the meta‐temporal composite (MTC) was averaged across amygdala, parahippocampal gyrus, temporal fusiform cortex, inferior‐temporal, and middle‐temporal bilateral ROIs. Gaussian mixture modeling was applied to baseline SUVR to establish tau positivity (T+) thresholds (MK‐6240 SUVR>1.39; FTP SUVR>1.36) 2SD above the mean of the lower distribution. Ten‐fold cross validation was used to characterize forward and backward SUVR prediction and T+/‐ stratification accuracy. Forward prediction was evaluated by predicting SUVR at each person’s last observation inputting each person’s first observation whereas backward prediction used the last observation to predict SUVR at the first observation. Results were compared with SILA validation using UC‐Berkeley processed ADNI Flortaucipir data with similar processing methods.

**Result:**

SILA exhibited similar prediction accuracy and midpoint conversion error in Wisconsin and ADNI studies. Figure 1B/D depicts MTC‐tau onset‐age‐aligned trajectories in Wisconsin and ADNI cohorts, respectively. Backward SUVR prediction error was lower and T+/‐ stratification accuracy was higher than forward prediction (ADNI RMSEforward=0.12, ADNI RMSEbackward=0.08; WI RMSEforward=0.22, WI RMSEbackward=0.17; forward accuracy=0.90, 0.92; backward prediction accuracy=0.96, 0.98 for ADNI and WI, respectively; Table 1). In the T‐ to T+ converters subset, backwards ETOA confidence intervals were within ‐2.68 to 2.04 years of midpoint between the observed last negative and first positive scan (Figure 2).

**Conclusion:**

SILA provides replicable estimates of T+ onset age and similar prediction accuracy with MK‐6240 and FTP. Future work will investigate how sources of tau PET variability influence temporal modeling outcomes.